# Anticancer activity of halofuginone in a preclinical model of osteosarcoma: inhibition of tumor growth and lung metastases

**DOI:** 10.18632/oncotarget.3891

**Published:** 2015-05-07

**Authors:** Audrey Lamora, Mathilde Mullard, Jérôme Amiaud, Régis Brion, Dominique Heymann, Françoise Redini, Franck Verrecchia

**Affiliations:** ^1^ INSERM, UMR 957, Equipe Labellisée Ligue contre le Cancer 2012, Nantes, France; ^2^ Université de Nantes, Laboratoire de Physiopathologie de la Résorption Osseuse et Thérapie des Tumeurs Osseuses Primitives, Nantes, France; ^3^ CHU Hôtel Dieu, Nantes, France; ^4^ Inserm Liliane Bettencourt School, France

**Keywords:** halofuginone, osteosarcoma, TGF-β, metastases, bone remodeling

## Abstract

Osteosarcoma is the main malignant primary bone tumor in children and adolescents for whom the prognosis remains poor, especially when metastases are present at diagnosis. Because we recently demonstrated that TGF-β/Smad cascade plays a crucial role in osteosarcoma metastatic progression, we investigated the effect of halofuginone, identified as an inhibitor of the TGF-β/Smad3 cascade, on osteosarcoma progression. A preclinical model of osteosarcoma was used to evaluate the impact of halofuginone on tumor growth, tumor microenvironment and metastasis development. *In vivo* experiments showed that halofuginone reduces primary tumor growth and lung metastases development. *In vitro* experiments demonstrated that halofuginone decreases cell viability mainly by its ability to induce caspase-3 dependent cell apoptosis. Moreover, halofuginone inhibits the TGF-β/Smad3 cascade and the response of TGF-β key targets involved in the metastases dissemination process such as MMP-2. In addition, halofuginone treatment affects the “vicious cycle” established between tumor and bone cells, and therefore the tumor-associated bone osteolysis. Together, these results demonstrate that halofuginone decreased primary osteosarcoma development and associated lung metastases by targeting both the tumor cells and the tumor microenvironment. Using halofuginone may be a promising therapeutic strategy against tumor progression of osteosarcoma specifically against lung metastases dissemination.

## INTRODUCTION

Osteosarcoma, the most common primary malignant bone tumor in children and adolescents (median age of diagnosis: 18 years) [1], represents the third highest cause of cancer-related death in this age group [2]. The survival rate that reaches approximately 50–70% at 5 years depending on the series have unfortunately remained unchanged during the last few decades [3]. The standard treatment of osteosarcoma consists of complete surgical resection associated with neoadjuvant and adjuvant chemotherapy composed of several agents such as doxorubicin, cisplatin, methotrexate or ifosfamide [4]. Radiotherapy is rarely used due to the radiation resistance of this type of tumor. Many patients die from metastatic diseases, lungs being the most common metastatic site for osteosarcoma [5]. The lack of response to conventional treatment in osteosarcoma patients and the poor prognosis for the metastatic forms of the pathology underscore the urgency to develop novel therapeutic strategies.

Alterations of bone remodeling associated with osteosarcoma development play a central role in the progression of this disease. Osteosarcoma is thus characterized by abnormal osteoid bone formation and tumor-associated bone osteolysis which correlates with poor prognosis [6] and plays a crucial role in the establishment of a vicious cycle between the bone and cancer cells [7]. Briefly, the tumor cells secrete several factors able to induce, directly or indirectly the differentiation and maturation of osteoclasts leading to bone osteolysis [8, 9]. In turn, this osteoclastic bone destruction allows the release of growth factors stored in mineralized bone matrix which stimulate cancer cells and thus the tumor growth [7–9].

One of these factors stored in the bone matrix and released during the tumor-associated bone osteolysis is TGF-β1, a member of the TGF-β family. Although the role of TGF-β in cancer is complex, it is commonly accepted that during the later stages of disease, the TGF-β cascade promotes tumor progression mainly by its ability to stimulate epithelial to mesenchymal transition (in the context of carcinoma), tumor invasion, metastatic dissemination and/or escape from the immune system [10]. Concerning osteosarcoma, we recently demonstrated that TGF-β1 levels are higher in the serum of patients compared to healthy donors and that TGF-β/Smad3 signaling pathway is activated in human biopsies. Second, we showed that blocking the TGF-β/Smad signaling pathway by overexpressing Smad7, a specific Smad inhibitor, slows down the growth of the primary tumor. This is mainly because Smad7 overexpression in tumor cells affects the “vicious cycle” established between tumor cells and bone cells by its ability to decrease osteoclast activity. Importantly, we showed that Smad7 overexpression in osteosarcoma cells or the treatment of mice with the TβRI inhibitor SD-208 inhibits the development of lung metastases demonstrating that the inhibition of TGF-β/Smad signaling pathway may represent a promising therapeutic strategy against tumor progression of osteosarcoma specifically against the development of lung metastases [11].

Halofuginone hydrobromide, 7-bromo-6-chloro-3-[3-(3-hydroxy-2-piperidinyl)-2-oxopropyl]-4(3H)-quinazolinone, is a derivative alkaloid from febrifugine isolated from a Chinese plant *Dichroa febrifuga*. Halofuginone was first identified as an inhibitor of type I collagen synthesis and is used as an anti-fibrotic drug [12]. In the context of cancer, halofuginone has recently showed its ability to reduce Kaposi sarcoma lesions in a completed phase II clinical trial in patients with AIDS-related Kaposi sarcoma [13]. In addition, by using different experimental models, halofuginone was shown to be effective against cancer progression for example, in the case of breast cancer [14], melanoma bone metastasis [15], myeloma [16], pancreas [17], Wilms tumor [18], hepatocellular carcinoma [19], bladder carcinoma [20], glioma [21] and prostate cancer [22].

Because the effects of halofuginone against cancer and fibrosis diseases are mainly associated with its ability to inhibit the TGF-β signaling pathway and because we demonstrated that blocking TGF-β is a promising therapeutic strategy against tumor progression of osteosarcoma, the aim of this work was to evaluate the effect of halofuginone on osteosarcoma progression and metastatic development.

## RESULTS

### Halofuginone inhibits *in vivo* tumor growth

**Figure 1:**
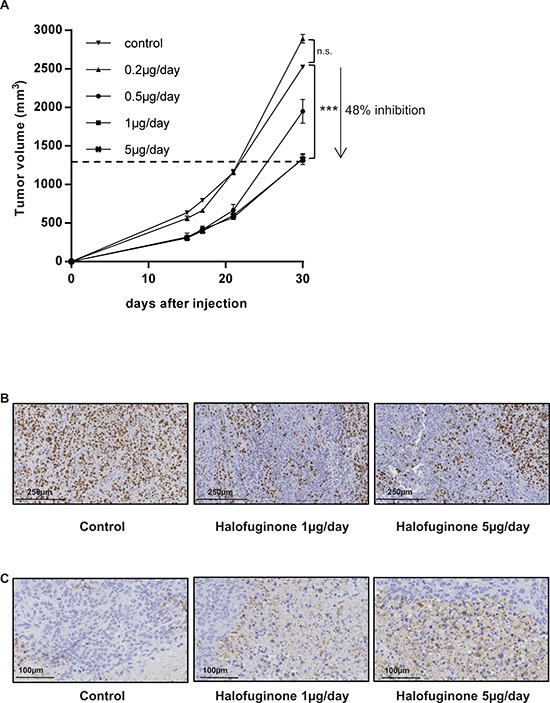
Halofuginone inhibits osteosarcoma primary tumor growth **A.** Mice were injected with 2.10^6^ HOS cells in each group. One day after cell injection, mice were daily intraperitoneal injected with 0.2 μg/mouse (*n* = 4), 0.5 μg/mouse (*n* = 4), 1 μg/mouse (*n* = 10) or 5 μg/mouse (*n* = 10) halofuginone or with vehicle (control group, *n* = 10). The results are representative of 2 independent experiments. The mean tumor volumes were calculated from day 1 to day 30. (Mean ± SEM; ***p < 0.005). **B.** Tumor samples (tumor sizes around 500 mm^3^) were fixed, embedded in paraffin, sectioned and stained with Ki-67. Representative photomicrographs per group are shown. **C.** Tumor samples (tumor sizes around 500 mm^3^) were fixed, embedded in paraffin, sectioned and stained with Caspase-3. Representative photomicrographs per group are shown.

To investigate the effect of halofuginone on osteosarcoma progression, we firstly used a mice preclinical experimental model of osteosarcoma induced by paratibial injection of HOS cells that mimics the human disease. As shown in Fig. 1A, the treatment of mice with halofuginone significantly inhibited the tumor growth in dose dependent manner. The mean tumor size at day 30 was 2524.3 ± 12.5 mm^3^, 2891.1 ± 54.5 mm^3^, 1949.8 ± 154.5 mm^3^, 1336.6 ± 50.8 mm^3^ and 1329.1 ± 70.1 mm^3^ when the mice were treated respectively with vehicle (control group) or with 0.2 μg/day, 0.5 μg/day, 1 μg/day or 5 μg/day of halofuginone. In this context, immunohistochemical staining for the proliferative marker Ki67 in mice tumor samples showed that halofuginone treatment decreases tumor cell proliferation (Fig. 1B) during the first stage of tumor development (tumor sizes around 500 mm^3^). In addition, immunohistochemical staining for the apoptotic marker caspase-3 in the same samples showed that the treatment of mice with halofuginone increases tumor cell apoptosis (Fig. 1C).

Together, these results demonstrate that the treatment of mice with halofuginone reduces *in vivo* tumor growth and suggest that this effect is mainly due to a decrease of tumor cell proliferation associated with an increase of cell death.

### Halofuginone induces *in vitro* cell death

**Figure 2:**
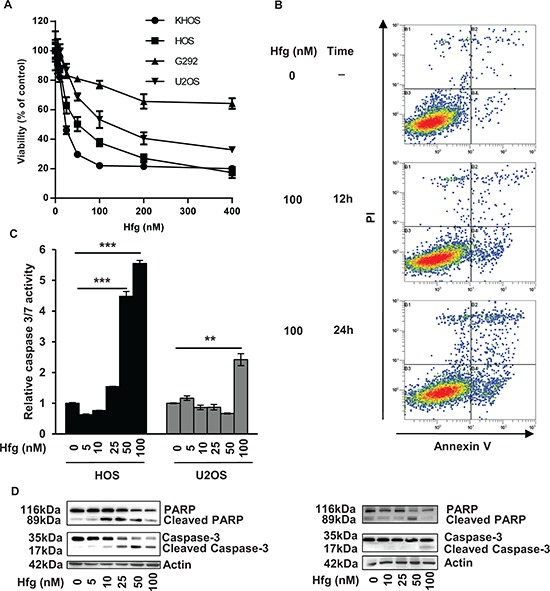
Halofuginone induces osteosarcoma cell death *in vitro* **A.** Four osteosarcoma cell lines were treated for 24 hours with halofuginone as indicated. After incubation, cell viability was evaluated by WST-1 test. For each cell line, graph indicates the percentage of cell viability compared to untreated cells. Mean ± S.D. of two independent experiments, each performed in sixplicate is presented. **B.** Representative dot plots of untreated HOS cells and HOS cells treated with 100 nM halofuginone for 12 and 24 hours are shown (Representative graphs of two experiments). **C.** HOS and U2OS cells were treated with halofuginone as indicated for 24 hours. Relative caspase-3/7 activity was evaluated. Bars indicate the caspase-3/7 activity for each halofuginone concentration tested (mean ± S.D.) of at least two independent experiments, each performed in triplicate (***p < 0.005, **p < 0.01). **D.** HOS cells (left panels) and U2OS cells (right panels) were treated with halofuginone as indicated for 24 hours. After incubation, Caspase-3 and PARP levels were detected by Western Blot analysis of whole cell lysates. Actin antibody was used as internal control. Representative blots of three experiments were shown.

To better understand the mechanisms involved in halofuginone-induced inhibition of osteosarcoma tumor growth, we next performed *in vitro* experiments. We firstly demonstrated that halofuginone significantly inhibits the viability of four osteosarcoma cell lines in a dose dependent manner after 24 h incubation (Fig. 2A). We secondly studied whether the reduced survival of osteosarcoma cells by halofuginone was associated with apoptosis induction. Flow cytometric annexin V/PI assay showed that halofuginone induces an early and late apoptotic cell populations in a dose-and time-dependent manner (Fig. 2B). The percentage of cells in early apoptosis (AnnexinV+/PI-) reached 0.62% in the absence of halofuginone and reached 3.05% and 5.37% after respectively 12 h and 24 h of cell treatment with 100 nM halofuginone. Similarly, the percentage of cells in late apoptosis (AnnexinV+/PI+) reached 0.69% in the absence of halofuginone and reached 1.08% and 3.82% after respectively 12 h and 24 h of treatment with 100 nM halofuginone. Thirdly, caspases-3/7 activity was measured in HOS and U2OS cells after 24 h incubation with halofuginone. As shown in Fig. 2C, halofuginone stimulated caspase-3/7 activity in a dose dependent manner. Finally, western immunoblotting analysis demonstrated the cleavage of caspase-3 and PARP in a dose dependent manner in HOS and U2OS cell lines (Fig. 2D).

Together, these results show that halofuginone inhibits cell viability *in vitro* and suggest that this effect is mainly due to a decrease of tumor cell proliferation possibly associated with an increase in cell death.

### Halofuginone inhibits tumor associated bone osteolysis

**Figure 3:**
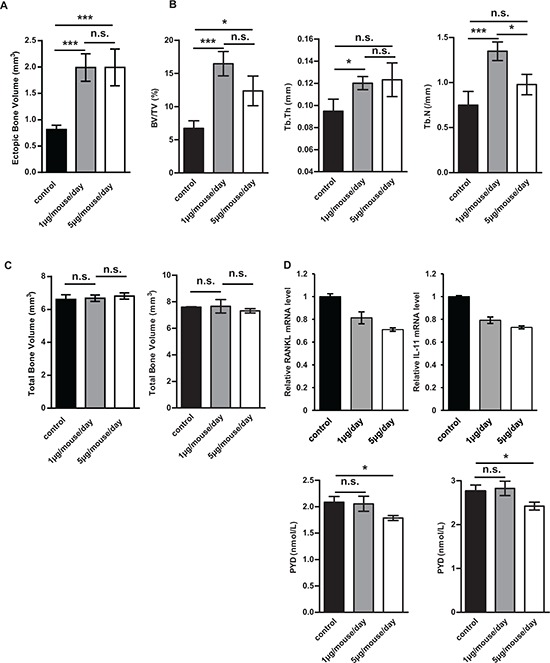
Halofuginone affects tumor associated bone remodeling Paratibial injections of 2.10^6^ HOS tumor cells were performed in 3 groups of nude mice (vehicle, halofuginone 1 μg/mouse/day and 5 μg/mouse/day). Bone remodeling parameters were analysed on the tibia bearing tumor (A–B). **A.** Histogram represents the ectopic bone volume (mean ± S.E.M.) when the tumor volume reached 2500 mm^3^ in each group of 10 mice (treated with 1 μg/day/mouse or 5 μg/day/mouse halofuginone or vehicle) (***p < 0.005). **B.** Left panel : histogram represents the specific trabecular bone volume (mean ± S.E.M.) when the tumor volume reached 1000 mm^3^ in each group of 6 mice (treated with 1 μg/day/mouse or 5 μg/day/mouse halofuginone or vehicle) (***p < 0.005, *p < 0.05). Middle panel : Bars indicate the trabecular thickness (mean ± S.E.M.) in each group (*p < 0.05). Right panel : Bars indicate the trabecular number (mean ± S.E.M.) in each group (***p < 0.005, *p < 0.05). **C.** Left panel : Bone volumes of healthy controlateral legs were measured when the tumor volume reached 2500 mm^3^ (left panel). Histograms represent the mean ± S.E.M. in each group. Right panel : Bone volumes of healthy contralateral legs were measured 35 days after HOS cell injection. Histograms represent the mean ± S.E.M. in each group. **D.** Upper panels : RNA was extracted from tumor biopsies of mice treated with 1 μg/mouse/day, or 5 μg/mouse/day halofuginone or vehicle. *RANKL* (left panel) and *IL-11* (right panel) mRNA steady-state levels were determined by quantitative RT-PCR. Bars indicate means ± S.D. Lower panels : Concentrations of pyridinoline in mice serum of control, 1 μg/mouse/day and 5 μg/mouse/day halofuginone groups were measured using the MicroVue Serum PYD EIA kit both when tumor volume reached 1000 mm^3^ (left panel) and 2500 mm^3^(right panel). Bars indicate means ± S.E.M performed in duplicate (*p < 0.05).

Because osteosarcoma-associated alteration of bone remodeling plays a central role in the development and progression of primary bone tumors, we evaluated the ability of halofuginone to alter tumor-associated bone remodeling. To this aim, the bone microarchitecture was examined in mice bearing HOS tumors using a high-resolution X-ray micro-CT system. First, to evaluate the ability of halofuginone to affect the production of bone, we specifically quantified ectopic bone formation. As shown in Fig. 3A, the ectopic bone volume in mice treated with 1 μg/day and 5 μg/day halofuginone was significantly higher than in mice injected with vehicle (1.99 ± 0.06 mm3 and 1.99 ± 0.08 mm3 vs. 0.81 ± 0.02 mm3 respectively, *p* < 0.005). We then analyzed the ability of halofuginone to alter bone osteolysis by evaluating the specific trabecular bone volume (BV/TV), trabecular number (Tb.N) and trabecular thickness (Tb.Th) when the tumor sizes reached 1000 mm^3^. As shown in Fig. 3B, control mice had lower trabecular bone volume than mice treated with 1 μg/day and 5 μg/day (6.72 ± 0.43% vs. 16.45 ± 0.68% and 12.37 ± 0.83%, *p* < 0.005 and *p* < 0.05 respectively). Moreover, control mice had a significant lower Tb.Th than mice treated with for example 1 μg/day halofuginone (0.095 ± 0.004 mm vs. 0.120 ± 0.002 mm, *p* < 0.05). Similarly, the Tb.N was also lower in the control group compared to the 1 μg/day halofuginone treated group (0.75 ± 0.06 /mm vs. 1.35 ± 0.04 /mm; *p* < 0.005). Similar results are observed when tumor volumes reached around 500 mm^3^ (data not shown). Interestingly, systemic treatment of mice with halofuginone does not significantly affect bone volume in the absence of tumor (Fig. 3C). To understand the effect of halofuginone on bone osteolysis, we next analyzed the expression of two osteolytic genes, *RANKL* and *IL-11*, in tumor biopsies from mice. qRT-PCR analysis indicated that bone tumors from mice treated with 1 μg/day and 5 μg/day halofuginone expressed significantly lower mRNA levels of *RANKL* and *IL-11* than control groups (Fig. 3D, upper panel). Finally, halofuginone treatment inhibits the degradation of collagen evaluated by the measure of pyridinoline excretion in mice serum (Fig. 3D, lower panel) both when tumor volumes reached 1000 mm^3^ (left panel) and 2500 mm^3^ (right panel).

Together these results demonstrate that halofuginone both decreased tumor-induced bone osteolysis and promoted tumor-induced bone formation, and suggest that these effects are partly due to the ability of halofuginone to reduce the expression of *RANKL* and *IL-11* by tumors cells.

### Treatment of mice with halofuginone inhibits the dissemination of pulmonary metastases

**Figure 4:**
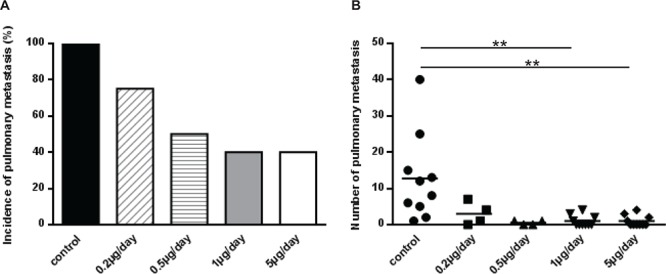
Treatment of mice with halofuginone inhibits lung metastases development Mice were injected with HOS cells and treated with vehicle or halofuginone as indicated. Mice were sacrificed when tumor sizes reached 2500 mm^3^ and lungs were removed. **A.** Histogram indicates lung metastases incidence in each group. **B.** Graph indicates individual (dots) and mean (lines) numbers of lung metastases counted in mice lungs from each group (**p < 0.01).

To evaluate the effect of halofuginone on pulmonary metastases development, the lungs of mice were removed when primary bone tumor volumes reached 2500 mm^3^. As shown in Figure 4, halofuginone reduced the incidence of lung metastases. In the absence of halofuginone treatment, 10 mice out of 10 (100%) developed lung metastases. By contrast, only 3 of 4 (75%) mice treated with 0.2 μg/day halofuginone, 2 of 4 (50%) mice treated with 0.5 μg/day halofuginone and 4 out of 10 (40%) mice treated with 1 μg/day or 5 μg/day halofuginone developed lung metastases (Fig. 4A). As shown in Fig. 4B, the treatment of mice with respectively 0.2 μg/day, 0.5 μg/day, 1 μg/day and 5 μg/day halofuginone also diminished the number of lung metastases when lungs are infected. No mice in the 5 μg/day and 1 μg/day halofuginone treated groups developed more than 5 lung metastases. By contrast, 8 mice in the control group developed more than 5 lung metastases.

Together, these results prove that halofuginone reduces both the incidence and the number of lung metastases.

### Halofuginone blocks the TGF-β/Smad3 signaling pathway in osteosarcoma cells

**Figure 5:**
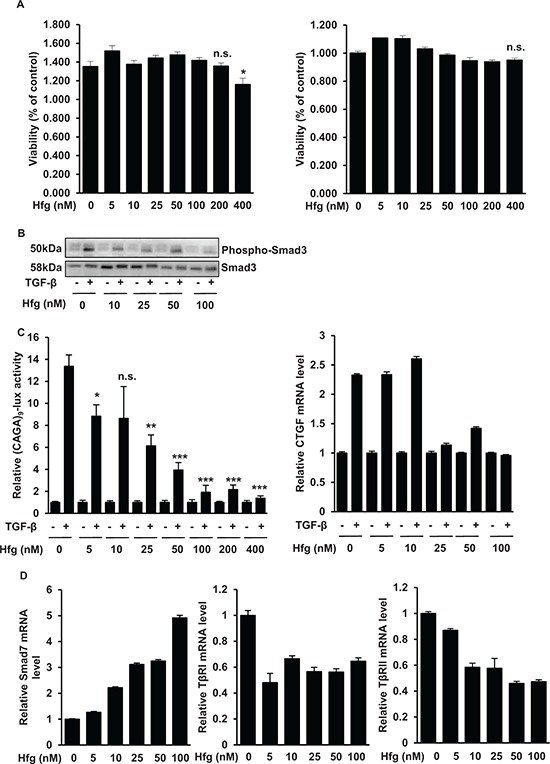
Halofuginone blocks the TGF-β/Smad3 cascade in osteosarcoma cells **A.** HOS (left panel) and U2OS (right panel) cells were treated with halofuginone as indicated for 10 hours. After incubation, cell viability was evaluated by WST-1 test. For each cell line, histogram indicates the percentage of cell viability compared to untreated cells (Mean ± S.D. of two independent experiments, each performed in sixplicate; *p < 0.05). **B.** HOS cells were treated for 4 hours with halofuginone and then for 30 min in the presence or absence of TGF-β (5 ng/ml). Phospho-Smad3 levels were detected by Western Blot analysis of HOS whole cell lysates (upper panel). The specificity of the modulation was confirmed with an anti-Smad3 antibody (lower panel). **C.** Left panel : HOS cells were transfected with the Smad3/4-specific construct (CAGA)_9_-luc. 24 h after transfection, cells were treated with halofuginone for 4 hours and then for 6 hours in the presence or absence of TGF-β (5 ng/ml). Bars indicate mean ± S.D. of at least two independent experiments carried out in triplicate (***p < 0.005, **p < 0.01, *p < 0.05). Right panel : HOS cells were treated with halofuginone for 4 hours and then for 6 hours in the presence or absence of TGF-β (5 ng/ml). After incubations, mRNA steady-state levels of the specific TGF-β target genes *CTGF* was determined by quantitative RT-PCR. Bars indicate means ± S.D. of at least two independent experiments, each performed in triplicate. **D.** HOS cells were treated or not with halofuginone during 10 hours as indicated. After incubations, mRNA steady-state levels of *Smad7* (left panel), *TβRI* (middle panel) and *TβRII* (right panel) were determined by quantitative RT-PCR. Bars indicate means ± S.D. of at least two independent experiments, each performed in triplicate.

Because halofuginone is known to reduce TGF-β signaling pathway in normal cells such as fibroblast [23] and in cancer cells such as melanoma [15] and promyelocytic leukemia [24], and because we recently demonstrated that TGF-β signaling plays a crucial role in the development of lung metastases in osteosarcoma, we then evaluated the effect of halofuginone on TGF-β signaling pathway in osteosarcoma cells. Firstly, we demonstrated that the incubation of HOS and/or U2OS cells with halofuginone during 10 h do not affect cell viability (Fig. 5A). In this context, to specifically evaluate the effect of halofuginone on TGF-β signaling, we chose to treat the cells only during 10 h, *ie* under experimental conditions which do not affect osteosarcoma cell viability. Halofuginone inhibits the ability of TGF-β to induce the phosphorylation of Smad3 (Fig. 5B), to transactivate the Smad3/4-specific reporter construct (CAGA)_9_-luc (Fig. 5C, left panel), and to stimulate the expression of CTGF (Fig. 5C, right panel), a specific TGF-β signaling pathway target gene, in HOS cells. Similar results were obtained with the U2OS cell line (Supplementary Fig. S1). To better understand by which mechanism halofuginone inhibits TGF-β signaling pathway, we investigated the effect of halofuginone on *Smad7*, *TβRI* and *TβRII* expressions. As indicated in Fig. 5D, halofuginone both induces *Smad7* expression, and inhibits *TβRI* and *TβRII* expressions even at very low doses (5 nM).

Together, these results demonstrate that halofuginone inhibits TGF-β signaling pathway probably by its ability to increase *Smad7* expression and to reduce the expression of the TGF-β receptors, *TβRI* and *TβRII*.

### Halofuginone inhibits TGF-β key targets involved in the metastatic process

**Figure 6:**
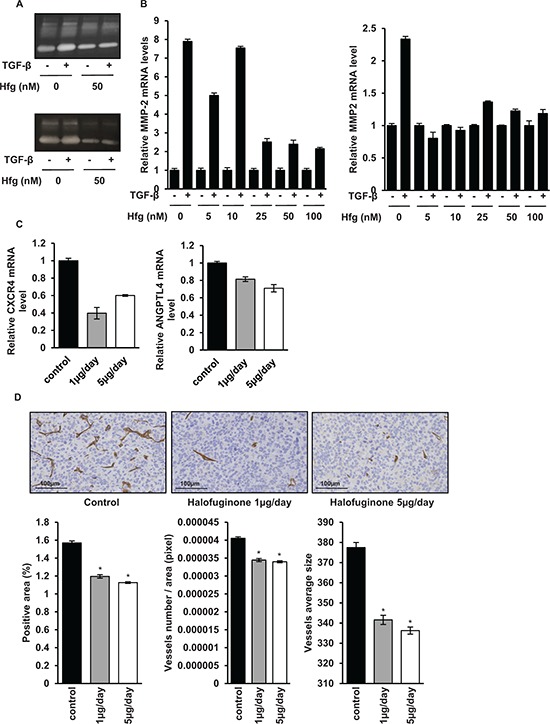
Halofuginone inhibits metastatic process **A.** Zymography analysis of conditioned media from 10 h serum-free cultures of HOS (upper panel) and U2OS (lower panel) cells treated with halofuginone for 4 hours and then for 6 h in the presence or absence of 5 ng/mL TGF-β. A Coomassie blue stained gel representative of three independent experiments is shown. **B.** HOS (left panel) and U2OS (right panel) cells were treated with halofuginone for 4 hours and then for 6 hours in the presence or absence of TGF-β1 (5 ng/ml). After incubation, *MMP-2* mRNA steady-state levels were determined by quantitative RT-PCR. Bars indicate mean ± S.D. of at least two independent experiments carried out in triplicate. **C.** RNA was extracted from tumor biopsies of mice treated with 1 μg/mouse/day, 5 μg/mouse/day halofuginone or vehicle. *CXCR4* (left panel) and *ANGPTL4* (right panel) mRNA steady-state levels were determined by quantitative RT-PCR. Bars indicate means ± S.D. **D.** Tumor samples (tumor sizes at 500 mm^3^) from mice treated or not with halofuginone as indicated were fixed, embedded in paraffin, sectioned and stained with CD146. Representative photomicrographs per group for HOS osteosarcoma mice are shown. Histograms represent the percentage of positive area for CD146 (left panel), the number of vessels per total area (in pixel) (middle panel) and the average size of vessels (right panel) in each group of mice (Mean ± SEM; *p < 0.05).

Since halofuginone is able to inhibit the TGF-β cascade in osteosarcoma cells, we evaluated the ability of halofuginone to inhibit TGF-β key targets involved in the metastatic process. As shown in Fig. 6A, zymography assay demonstrated that halofuginone strongly reduces the activation of MMP-2 in osteosarcoma cells, a metalloproteinase that plays a crucial role in the cell invasion process. Moreover, as shown in Fig. 6B, RT-qPCR analysis indicated that halofuginone inhibits the ability of TGF-β to induce *MMP-2* expression. In addition, RT-qPCR analysis from mice biopsies indicated that the expression by tumor cells of *CXCR4* and *ANGPTL4*, two TGF-β target genes identified as key players to prime cancer cells towards the lungs, were both reduced when mice were treated with halofuginone (Fig. 6C). Finally, immunohistochemical staining for the endothelial marker CD146 in mice tumor samples showed that halofuginone treatment significantly decreased the angiogenic process compared to the untreated group by decreasing the number of vessels and their average size (Fig. 6D).

Together, these results prove that halofuginone inhibits TGF-β key targets involved in the metastatic process of osteosarcoma.

## DISCUSSION

Currently a variety of agents appear to be of potential clinical interest for osteosarcoma, with an emphasis on molecular targeted approaches of signaling pathways [25–28]. Basic research thus identified specific targets and developed adapted strategies for osteosarcoma tumor inhibition. However, many of these therapeutic strategies focus on a single aspect of osteosarcoma tumor development and target only one point of the ‘vicious circle’ between the tumor cells and bone cells, either the tumor cells or the tumor environment. These strategies have thus attempted to either induce apoptosis and/or cell cycle arrest of tumor cells or to target the tumor microenvironment by the inhibition of osteoclast-mediated bone destruction for example. During the last decade, new strategies have associated the use of chemotherapeutic drugs that target tumor cells and drugs that target osteoclasts allowing a better response to treatment. Future directions and challenges could be the discovery of drugs able to target several key points of the ‘vicious circle’ established between tumor cells and bone cells in order to limit treatment escape.

In this study, we demonstrated that halofuginone inhibits tumor growth in human osteosarcoma xenograft model *in vivo*. To understand whether the tumor growth inhibition observed *in vivo* was due to the inhibition of osteosarcoma cell proliferation, to the induction of tumor cell apoptosis and/or to the drug effect on cell microenvironment, we have first tested the direct potential effect of halofuginone on osteosarcoma cell viability using WST-1 assay. As previously described in other tumor cell lines such as multiple myeloma or melanoma cell lines [15, 16], we demonstrated that all the four osteosarcoma cell lines tested are sensitive to halofuginone. In addition, annexin V/PI double staining showed that halofuginone induced apoptosis in a dose- and time- dependent manner. How halofuginone modulates intracellular signaling cascade leading to apoptosis is not fully understood. Signaling pathways that are able to induce apoptosis have been schematically classified into i) extrinsic pathways initiated by death receptors and ii) intrinsic pathways initiated by mitochondrial events [29]. Here, we demonstrated that halofuginone induced the cleavage and activation of caspase-3 a common mediator of both apoptotic pathways. In addition, we demonstrated that halofuginone induced the cleavage of PARP, a substrate of caspase-3 known as an indicator of DNA damage and apoptosis. Whatever the exact involvement of the intrinsic and/or extrinsic pathways, we clearly demonstrated that halofuginone induces *in vitro* and *in vivo* osteosarcoma cell death and thus inhibits the *in vivo* tumor growth.

We next focused our attention on the effects of halofuginone on tumor cell microenvironment, specifically on bone remodeling. In this study, using microCT analysis, we demonstrated that halofuginone inhibits the tumor-associated bone destruction by both promoting ectopic bone formation and preventing trabecular bone osteolysis. As first hypothesis, our results suggest that halofuginone effects on bone remodeling could be explained indirectly by the death of tumor cells induced by halofuginone. Since some studies showed that tumor cells produce osteoclast-activating factors which induce osteoclast precursor differentiation and activation [30, 31], the consequence of the decrease of the tumor cell number may be a diminution of the production and the release of osteolytic factors produced by tumor cells thus contributing to decreased osteoclast activity. As second hypothesis, our results suggest that halofuginone may act on bone remodeling by its ability to block the TGF-β signaling pathway. As previously described in many cancer cells such as melanoma [15], we here demonstrated that halofuginone inhibits TGF-β/Smad3 signaling pathway by increasing *Smad7* expression and/or reducing *TβRI* and *TβRII* expressions. In this context, we demonstrated in tumor biopsies that halofuginone inhibits the expression of *RANKL* and *IL-11* mRNA, two TGF-β target-cytokines that play a central role in bone osteolysis [30–32]. In addition, we recently demonstrated that blocking TGF-β signaling by overexpression of Smad7 in osteosarcoma cells inhibits the expression and release of osteolytic factors such as RANKL and IL-11 by tumor cells [11]. Finally, although the systemic treatment of mice with halofuginone did not show significant results on bone remodeling in the absence of tumor, we cannot rule out that halofuginone acts directly on osteoblast and or osteoclast activity. Here, we clearly demonstrated that halofuginone inhibits bone osteolysis associated to the tumor growth, which represents the first step of tumor development.

Finally, we have demonstrated that halofuginone inhibits the development of lung metastases. We recently showed that TGF-β signaling pathway plays a key role in the establishment and progression of osteosarcoma lung metastases. Indeed, overexpression of the Smad inhibitor Smad7, or the systemic injection of SD208, an inhibitor of TβRI kinase, inhibits the TGF-β signaling cascade and thus reduces the expression of TGF-β-regulated metastatic genes and the ability of TGF-β to stimulate migration and invasion of osteosarcoma cells [11]. Here, we demonstrated that the ability of halofuginone to inhibit the TGF-β/Smad3 cascade in osteosarcoma reduces the expression and activity of MMP-2, a protein highly implicated in the migration and invasion processes [11, 33, 34]. Interestingly, halofuginone was also able to inhibit the ability of TGF-β to stimulate *ANGPTL4* and *CXCR4* expression, two factors identified as key players to prime cancer cells for metastasis respectively towards the lungs [35] and towards the bones or the lungs [36]. Another major step in the metastatic process is the ability of TGF-β to stimulate tumor associated angiogenesis and thus the dissemination of tumor cells into the bloodstream [10]. In this context, we clearly demonstrated that halofuginone reduces the angiogenic process as shown by decreased immunohistochemical staining for the endothelial marker CD146, as previously reported with the inhibitor SD-208 [11].

To sum up, our data demonstrated that halofuginone decreased primary osteosarcoma development and associated lung metastases by targeting both the tumor cells and the bone remodeling. In this context, halofuginone represents a promising therapeutic drug that can act at different key points of osteosarcoma progression: on tumor cells by inducing their apoptosis, on tumor microenvironment by inhibiting tumor associated osteolysis and on the metastatic process mainly by inhibiting the TGF-β signaling pathway.

## MATERIALS AND METHODS

### Cell culture and reagents

The human osteosarcoma cell lines MNNG/HOS (HOS, young female high-grade osteosarcoma from femur origin transformed *in vitro* by N-methyl-N′-nitro-N-nitrosoguanidine treatment), KHOS (derived from HOS by transformation using Kirsten murine sarcoma virus Ki-MSV), U2OS (young female osteosarcoma from tibia origin) and G292 (young female osteosarcoma) were purchased from the American Type Culture Collection (respectively CRL-1547™, CRL-1544™, HTB-96™, CRL-1423™) and cultured in DMEM (Dulbecco's Modified Eagle's Medium, Lonza, Basel, Switzerland) supplemented with 10% fetal bovine serum (HyclonePerbio, Bezons, France) in a humidified 5% CO2/air atmosphere at 37°C. All cell lines were passaged for less than 3 months and were authenticated by short tandem repeat (STR) profiling. TGF-β1 and halofuginone (halofuginone hydrobromide, vetranal™) were respectively from R&D System, Inc (Minneapolis, MN) and Sigma (St Quentin-Fallavier, France). Halofuginone has been diluted in DPBS (Dulbecco's Phosphate Buffer Saline, Biowhittaker).

### Osteosarcoma mouse model

Four-week-old female Rj:NMRI-nude mice (Elevages Janvier, Le Genest Saint Isle, France) were maintained under pathogen-free conditions at the Experimental Therapy Unit (Faculty of Medicine, Nantes, France) in accordance with the institutional guidelines of the French Ethical Committee (CEEA Pays de la Loire n°06; project authorization n°1281.01) and under the supervision of authorized investigators. The mice were anesthetized by inhalation of a combination of isoflurane/air (1.5%, 1 L/min) before receiving an intramuscular injection of 2.10^6^ HOS osteosarcoma cells in close proximity to the tibia, leading to a rapidly growing tumor in soft tissue with secondary contiguous bone invasion. One day after HOS cells injection, some mice received different doses either 0.2 μg/mouse/ day (10 μg/kg/day), 0.5 μg/mouse/day (25 μg/kg/day), 1 μg/mouse/day (50 μg/kg/day), 5 μg/mouse/day (250 μg/kg/day) of halofuginone or control vehicle (DPBS) by daily intraperitoneal injection. Note that the weight of each mouse was similar for each group at the beginning of the experiment and no weight loss was observed following treatment with halofuginone. The tumor volume (V) was calculated from the measurement of two perpendicular diameters using a caliper, according to the following formula: *V* = 0.5 × L × (S)^2^ as previously described [37]. Mice were sacrificed when the tumor volume reached 2500 mm^3^ for ethical considerations. Under these conditions, pulmonary metastases developed when primary tumor volumes were at least 2000 mm^3^.

### Histologic analysis and immunohistochemistry of mice tumor samples

Tumor tissues were embedded in paraffin and 3-μm sections were cut and stained for caspase-3, Ki-67 and CD146 using respectively rabbit polyclonal anti-caspase-3 (Cell Signaling), anti-Ki67 (Dako), anti-CD146 (Abcam) antibodies. Immunodetection was performed using DAB Substrate-Chromogen (Dako) and counterstained with hematoxylin. Quantification of relative caspase-3 positive surface, Ki-67 positive cells and CD146 immunostaining in tumor tissue were evaluated by ImageJ (NIH, Bethesda, MD) softwares.

### Proliferation assay

Osteosarcoma cell lines were plated in DMEM with 10% FBS and treated with halofuginone as indicated. Cell growth and viability were determined by using a WST-1 cell proliferation assay kit (Takara bio inc.). Absorbance of the samples was measured at 440 nm wave length with Victor^2^™ (Perkin Elmer, life sciences) ELISA reader after 2 hours incubation.

### Cell cycle analysis

Osteosarcoma cell lines were incubated in the absence or the presence of halofuginone for 24 h, trypsinized, washed twice and incubated in PBS containing 0.12% Triton X-100, 0.12 mM EDTA and 100 μg/mL ribonuclease A; 50 μg/mL propidium iodide was then added to each sample for 20 min at 4°C. Cell cycle distribution was analysed by flow cytometry (Cytomics FC500; Beckman Coulter, Roissy, France) based on 2N and 4N DNA content.

### Annexine-V assay

Osteosarcoma cells were cultured and treated with or without halofuginone for 12 h or 24 h. Cells were washed and resuspended in 1X binding buffer (BD pharmingen™, BD Biosciences). Cells undergoing apoptosis were identified by flow cytometry (Cytomics FC500; Beckman Coulter, Roissy, France) after 20 min incubation with annexin V-FITC and propidium iodide.

### Caspase 3/7 activity

Caspase 3/7 activity was measured using a Caspase-Glo assay kit (Promega). Briefly, cells were lysed in RIPA buffer (10 mM Tris pH8, 1 mM EDTA, 150 mM NaCl, 1% NP40, 0.1% SDS) containing a cocktail of protease and phosphatase inhibitors (1 mM sodium orthovanadate (Na_2_VO_4_), 1 mM phenylmethylsulforyl fluoride (PMSF), 10 mM sodium fluoride (NaF), 10 mM N-ethylmaleimide (NEM), 2 μg/ml leupeptin and 1 μg/ml pepstatine) at 4°C and protein concentration was determined by BCA kit (Sigma, St Quentin-Fallavier, France). An equal volume of reagents was added to a white-walled 96-well plate containing equal amounts of total protein extracts and incubated at room temperature for 1 h. The luminescence of each sample was measured in a plate-reading luminometer Tristar LB941 (Berthold technologies).

### Western blot analysis

Cells were lysed in RIPA buffer and protein concentration was determined as described above. Samples containing equal amounts of total protein extracts (depending on the antibody, 30–50 μg) in Laemmli buffer (62.5 mM Tris–HCl, pH 6.8, 2% SDS, 10% glycerol, 5% 2-mercaptoethanol, 0.001% bromophenol blue) were separated by SDS-polyacrylamide gel electrophoresis, and transferred to PVDF Transfer membrane (Thermo scientific). The membranes were blocked in 3% BSA–PBS-0.1% Tween at room temperature for 1 h and blots were probed overnight at 4°C with primary antibodies anti-phospho-Smad3 (Millipore, Temecula, CA, USA), anti-Smad3 (Millipore), anti-PARP (Cell signaling technology, Beverly, USA), anti-Caspase 3 (Cell Signaling Technology) or anti-β-actin (Sigma) antibodies. After incubation, the membranes were washed three times with washing buffer (PBS containing 0.1% Tween) for 5 min. Membranes were then incubated for 1 h with 1:10, 000 diluted secondary antibodies (Santa Cruz Biotechnologies, Santa Cruz, CA) at room temperature. Specific proteins were detected using G-Box (Syngene, Cambridge, UK) after washing. Antibody binding was visualized with the enhanced chemiluminescence system (SuperSignal West Pico Chemiluminescent Substrate, ThermoSientific, Illkirch, France).

### Micro-CT analysis

Analysis of bone microarchitecture was performed using the high-resolution X-ray micro-CT system for small-animal imaging SkyScan-1072 (SkyScan, Kartuizersweg, Belgium) at different tumor volumes (500, 1000 and 2500 mm^3^). All tibia/fibula were scanned using the same parameters (pixel size 18 μm, 50 kV, 0.5-mm aluminium filter and 0.6 degrees per rotation step). Analysis of bone parameters were performed using the CTan software (Skyscan). The bone volume (BV) of ectopic bone was quantified over the total length of tibia bearing 2500 mm^3^ tumor. Trabecular structures positioned 0.2 mm below the growth plate were quantified over a length of 1 mm at 1000 mm^3^ tumor volume since at higher tumor volume trabecular bone is completely destroyed by the tumor cells. Bone volume/trabecular volume (BV/TV), trabecular thickness (Tb.Th) and trabecular number (Tb.N) were determined.

### Real-time polymerase chain reaction

Total RNA from cell lines was extracted using NucleoSpin^®^RNAII (Macherey Nagel, Duren, Germany). Total RNA from tumors was extracted using the TRIzol reagent (Invitrogen Life Technologies) after mechanical grinding with Turrax (IKA, Staufen, Switzerland). Total RNA was reversed transcribed using the Maxima H minus first stand cDNA synthesis kit (Thermoscientific). Real-time monitoring of PCR amplification of complementary DNA was performed using DNA primers (primers sequences are available in Table 1) on CFX96 real-time PCR detector system (Bio-Rad, Marnes la Coquette, France) with SYBR PCR Master Mix buffer (Bio-Rad). Target gene expression was normalized to glyceraldehyde 3-phosphatedehydrogenase (GAPDH) levels in respective samples as an internal standard.

**Table 1:** Primer sequence for quantitative RT-PCR

	Sense	Antisense
*ANGPTL4*	gAC CCg gCT CAC AAT gTC	CCC TgA ggC Tgg ATT TCA
*CTGF*	CTC CTg Cag gCT AgA gAA gC	gAT gCA CTT TTT gCC CTT CTT
*CXCR4*	CCg Agg AAA Tgg gCT Cag ggg A	TgA Tgg AgT AgA Tgg Tgg gCA ggA
*GAPDH*	Tgg gTg TgA ACC Atg AgA AgT Atg	ggT gCA ggA ggC ATT gCT
*IL-11*	gCA gCg gAC Agg gAA ggg TTA A	ACA ggC TCA gCA CgA CCA gg
*MMP2*	AgA Agg CTg TgT TCT TTg CAg	Agg CTg gTC AgT ggC TTg
*RANKL*	TCg TTg gAT CAC AgC ACA TCA	TCg TTg gAT CAC AgC ACA TCA
*Smad7*	TTT gCC TCg gAC AgC TCA AT	TTT TTg CTC Cgc ACC TTC Tg
*TβRI*	gCA gAC TTA ggA CTg gCA gTA Ag	AgA ACT TCA ggg gCC ATg T
*TβRII*	CCA CCA CCA ggg CAT CCA	TCg Tgg TCC CAg CAC TCA

### Collagen degradation

The degradation of collagen was evaluated by the measure of pyridinoline excretion in mice serum using the MicroVue Serum PYD EIA kit (Quidel, CA).

### Transient cell transfection, reporter assay and plasmid construct

Transient cell transfections were performed with jetPEI™ (Polyplus-transfection, Illkirch, France). The phRLMLP-*Renilla* luciferase expression vector was cotransfected in all experiments to monitor transfection efficiencies. Luciferase activity was determined with the Dual-Luciferase reporter assay system (Promega, Charbonnieres, France). The (CAGA)_9_-Luc construct was used as a reporter construct specific for Smad3/4-driven signaling.

### Gelatin zymography

Cells were cultured without serum for 10 h and their conditioned media were analyzed by gelatin zymography in 10% polyacrylamide gels containing 1 mg/ml gelatin (Sigma-Aldrich) as described previously [33].

### Statistical analysis

All analyses were performed using GraphPad Prism 4.0 software (GraphPad Software, La Jolla, CA, USA) or Excel. Results of *in vitro* experiments were analyzed with the unpaired *t*-test and are given as means ± SD. For *in vivo* experiments, results from groups treated with 1 μg/mouse/day or 5 μg/mouse/day were compared with no treated control groups using the unpaired *t*-test and are given as means ± SEM. Results with *p* < 0.05 were considered significant.

## SUPPLEMENTARY FIGURE



## References

[1] Ottaviani G, Jaffe N (2009). The epidemiology of osteosarcoma. Cancer Treat Res.

[2] Dass CR, Ek ET, Contreras KG, Choong PF (2006). A novel orthotopic murine model provides insights into cellular and molecular characteristics contributing to human osteosarcoma. Clin Exp Metastasis.

[3] Ando K, Heymann M-F, Stresing V, Mori K, Rédini F, Heymann D (2013). Current therapeutic strategies and novel approaches in osteosarcoma. Cancers.

[4] Anninga JK, Gelderblom H, Fiocco M, Kroep JR, Taminiau AHM, Hogendoorn PCW, Egeler RM (2011). Chemotherapeutic adjuvant treatment for osteosarcoma: where do we stand?. Eur J Cancer Oxf Engl 1990.

[5] Patel SJ, Lynch JW, Johnson T, Carroll RR, Schumacher C, Spanier S, Scarborough M (2002). Dose-intense ifosfamide/doxorubicin/cisplatin based chemotherapy for osteosarcoma in adults. Am J Clin Oncol.

[6] Clark JCM, Dass CR, Choong PFM (2008). A review of clinical and molecular prognostic factors in osteosarcoma. J Cancer Res Clin Oncol.

[7] Halvorson KG, Sevcik MA, Ghilardi JR, Rosol TJ, Mantyh PW (2006). Similarities and differences in tumor growth, skeletal remodeling and pain in an osteolytic and osteoblastic model of bone cancer. Clin J Pain.

[8] Guise TA, Yin JJ, Taylor SD, Kumagai Y, Dallas M, Boyce BF, Yoneda T, Mundy GR (1996). Evidence for a causal role of parathyroid hormone-related protein in the pathogenesis of human breast cancer-mediated osteolysis. J Clin Invest.

[9] Grano M, Mori G, Minielli V, Cantatore FP, Colucci S, Zallone AZ (2000). Breast cancer cell line MDA-231 stimulates osteoclastogenesis and bone resorption in human osteoclasts. Biochem Biophys Res Commun.

[10] Meulmeester E, Ten Dijke P (2011). The dynamic roles of TGF-β in cancer. J Pathol.

[11] Lamora A, Talbot J, Bougras G, Amiaud J, Leduc M, Chesneau J, Taurelle J, Stresing V, Le Deley MC, Heymann MF, Heymann D, Redini F, Verrecchia F (2014). Overexpression of smad7 blocks primary tumor growth and lung metastasis development in osteosarcoma. Clin Cancer Res Off J Am Assoc Cancer Res.

[12] Pines M, Nagler A (1998). Halofuginone: a novel antifibrotic therapy. Gen Pharmacol.

[13] Koon HB, Fingleton B, Lee JY, Geyer JT, Cesarman E, Parise RA, Egorin MJ, Dezube BJ, Aboulafia D, Krown SE (2011). Phase II AIDS Malignancy Consortium trial of topical halofuginone in AIDS-related Kaposi sarcoma. J Acquir Immune Defic Syndr 1999.

[14] Jin ML, Park SY, Kim YH, Park G, Lee SJ (2014). Halofuginone induces the apoptosis of breast cancer cells and inhibits migration via downregulation of matrix metalloproteinase-9. Int J Oncol.

[15] Juárez P, Mohammad KS, Yin JJ, Fournier PGJ, McKenna RC, Davis HW, Peng XH, Niewolna M, Javelaud D, Chirgwin JM, Mauviel A, Guise TA (2012). Halofuginone inhibits the establishment and progression of melanoma bone metastases. Cancer Res.

[16] Leiba M, Jakubikova J, Klippel S, Mitsiades CS, Hideshima T, Tai Y-T, Leiba A, Pines M, Richardson PG, Nagler A, Anderson KC (2012). Halofuginone inhibits multiple myeloma growth *in vitro* and *in vivo* and enhances cytotoxicity of conventional and novel agents. Br J Haematol.

[17] Spector I, Honig H, Kawada N, Nagler A, Genin O, Pines M (2010). Inhibition of pancreatic stellate cell activation by halofuginone prevents pancreatic xenograft tumor development. Pancreas.

[18] Pinthus JH, Sheffer Y, Nagler A, Fridman E, Mor Y, Genina O, Pines M (2005). Inhibition of Wilms tumor xenograft progression by halofuginone is accompanied by activation of WT-1 gene expression. J Urol.

[19] Nagler A, Ohana M, Shibolet O, Shapira MY, Alper R, Vlodavsky I, Pines M, Ilan Y (2004). Suppression of hepatocellular carcinoma growth in mice by the alkaloid coccidiostat halofuginone. Eur J Cancer Oxf Engl 1990.

[20] Elkin M, Miao HQ, Nagler A, Aingorn E, Reich R, Hemo I, Dou HL, Pines M, Vlodavsky I (2000). Halofuginone: a potent inhibitor of critical steps in angiogenesis progression. FASEB J Off Publ Fed Am Soc Exp Biol.

[21] Abramovitch R, Dafni H, Neeman M, Nagler A, Pines M (1999). Inhibition of neovascularization and tumor growth, and facilitation of wound repair, by halofuginone, an inhibitor of collagen type I synthesis. Neoplasia N Y N.

[22] Gavish Z, Pinthus JH, Barak V, Ramon J, Nagler A, Eshhar Z, Pines M (2002). Growth inhibition of prostate cancer xenografts by halofuginone. The Prostate.

[23] Xavier S, Piek E, Fujii M, Javelaud D, Mauviel A, Flanders KC, Samuni AM, Felici A, Reiss M, Yarkoni S, Sowers A, Mitchell JB, Roberts AB, Russo A (2004). Amelioration of radiation-induced fibrosis: inhibition of transforming growth factor-beta signaling by halofuginone. J Biol Chem.

[24] De Figueiredo-Pontes LL, Assis PA, Santana-Lemos BAA, Jácomo RH, Lima ASG, Garcia AB, Thomé CH, Araújo AG, Panepucci RA, Zago MA, Nagler A, Falcão RP, Rego EM (2011). Halofuginone has anti-proliferative effects in acute promyelocytic leukemia by modulating the transforming growth factor beta signaling pathway. PloS One.

[25] Chou AJ, Geller DS, Gorlick R (2008). Therapy for osteosarcoma: where do we go from here?. Paediatr Drugs.

[26] Hattinger CM, Pasello M, Ferrari S, Picci P, Serra M (2010). Emerging drugs for high-grade osteosarcoma. Expert Opin Emerg Drugs.

[27] Van Oosterwijk JG, Anninga JK, Gelderblom H, Cleton-Jansen A-M, Bovée JVMG (2013). Update on targets and novel treatment options for high-grade osteosarcoma and chondrosarcoma. Hematol Oncol Clin North Am.

[28] Grignani G, Palmerini E, Dileo P, Asaftei SD, D'Ambrosio L, Pignochino Y, Mercuri M, Picci P, Fagioli F, Casali PG, Ferrari S, Aglietta M (2012). A phase II trial of sorafenib in relapsed and unresectable high-grade osteosarcoma after failure of standard multimodal therapy: an Italian Sarcoma Group study. Ann Oncol Off J Eur Soc Med Oncol ESMO.

[29] Elmore S (2007). Apoptosis: a review of programmed cell death. Toxicol Pathol.

[30] Chirgwin JM, Guise TA (2000). Molecular mechanisms of tumor-bone interactions in osteolytic metastases. Crit Rev Eukaryot Gene Expr.

[31] Chirgwin JM, Mohammad KS, Guise TA (2004). Tumor-bone cellular interactions in skeletal metastases. J Musculoskelet Neuronal Interact.

[32] Boyle WJ, Simonet WS, Lacey DL (2003). Osteoclast differentiation and activation. Nature.

[33] Javelaud D, Delmas V, Möller M, Sextius P, André J, Menashi S, Larue L, Mauviel A (2005). Stable overexpression of Smad7 in human melanoma cells inhibits their tumorigenicity *in vitro* and *in vivo*. Oncogene.

[34] Javelaud D, Mohammad KS, McKenna CR, Fournier P, Luciani F, Niewolna M, André J, Delmas V, Larue L, Guise TA, Mauviel A (2007). Stable overexpression of Smad7 in human melanoma cells impairs bone metastasis. Cancer Res.

[35] Padua D, Zhang XH-F, Wang Q, Nadal C, Gerald WL, Gomis RR, Massagué J (2008). TGFbeta primes breast tumors for lung metastasis seeding through angiopoietin-like 4. Cell.

[36] Liang Z, Wu T, Lou H, Yu X, Taichman RS, Lau SK, Nie S, Umbreit J, Shim H (2004). Inhibition of breast cancer metastasis by selective synthetic polypeptide against CXCR4. Cancer Res.

[37] Heymann D, Ory B, Blanchard F, Heymann M-F, Coipeau P, Charrier C, Couillaud S, Thiery JP, Gouin F, Redini F (2005). Enhanced tumor regression and tissue repair when zoledronic acid is combined with ifosfamide in rat osteosarcoma. Bone.

