# Supplementary material for: Anticancer activity of halofuginone in a preclinical model of osteosarcoma: inhibition of tumor growth and lung metastases
Source: Oncotarget. 2015 May 7;6(16):14413–27. doi: 10.18632/oncotarget.3891 (PMC4546476; doi:10.18632/oncotarget.3891)
Supplement: Supplementary file 1 [file oncotarget-06-14413-s001.pdf]

SUPPLEMENTARY FIGURE

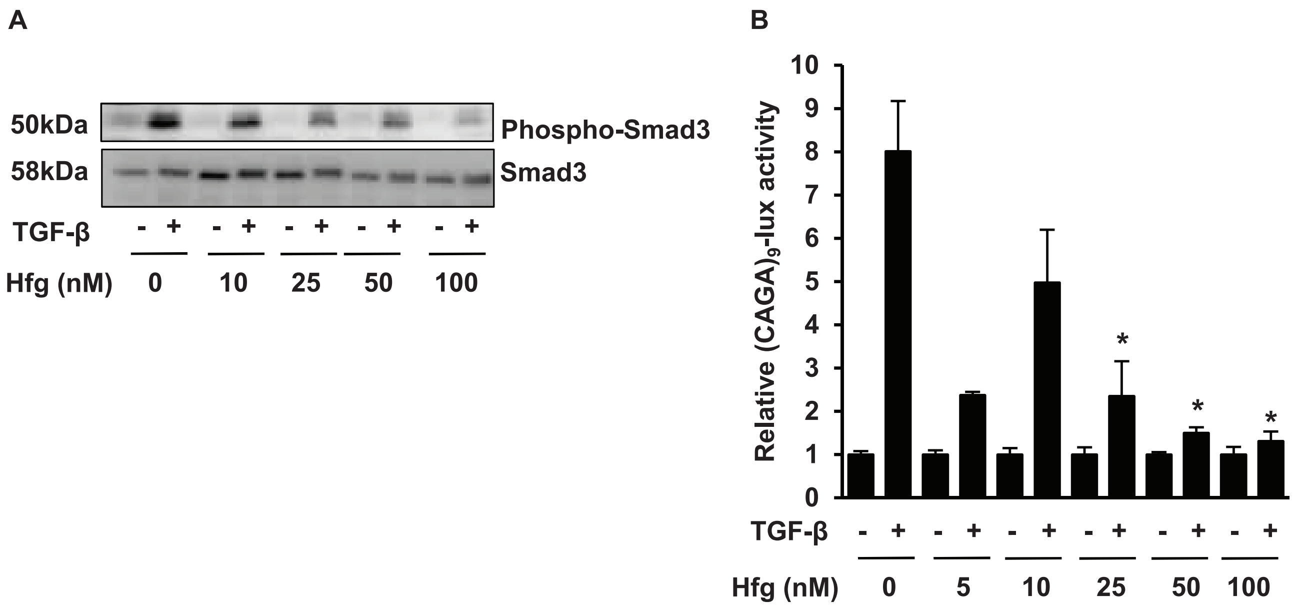

Supplementary Figure S1: Halofuginone blocks the TGF-β/Smad3 cascade in U2OS cells. A. U2OS cells were treated for 4 hours with halofuginone as indicated and then for 30 min in the presence or absence of TGF-β (5 ng/ml). Phospho-Smad3 levels were detected by Western Blot analysis of U2OS whole cell lysates (upper panel). Anti-Smad3 antibody was used as internal control (lower panel). **B.** U2OS cells were transfected with the Smad3/4-specific construct $(CAGA)_9$-luc. 24 h after transfection, cells were treated for 4 hours with halofuginone as indicated and then for 6 hours in presence or absence of TGF-β (5 ng/ml). Bars indicate mean ± S.D. of at least two independent experiments carried out in triplicate (*$p < 0.05$).
